# The Transferability of Spectral Grain Yield Prediction in Wheat Breeding across Years and Trial Locations

**DOI:** 10.3390/s23084177

**Published:** 2023-04-21

**Authors:** Lukas Prey, Ludwig Ramgraber, Johannes Seidl-Schulz, Anja Hanemann, Patrick Ole Noack

**Affiliations:** 1 Hochschule Weihenstephan-Triesdorf, Markgrafenstrasse 16, 91746 Weidenbach, Germany; 2 Saatzucht Josef Breun GmbH & Co. KG, Amselweg 1, 91074 Herzogenaurach, Germany; 3 Geo-Konzept GmbH, Wittenfelder Strasse 28, 85111 Adelschlag, Germany

**Keywords:** high-throughput phenotyping, digital breeding, phenomics, multispectral sensing, data fusion, date fusion, dataset effect, across-trials, non-destructive harvest

## Abstract

Grain yield (GY) prediction based on non-destructive UAV-based spectral sensing could make screening of large field trials more efficient and objective. However, the transfer of models remains challenging, and is affected by location, year-dependent weather conditions and measurement dates. Therefore, this study evaluates GY modelling across years and locations, considering the effect of measurement dates within years. Based on a previous study, we used a normalized difference red edge (*NDRE*1) index with PLS (partial least squares) regression, trained and tested with the data of individual dates and date combinations, respectively. While strong differences in model performance were observed between test datasets, i.e., different trials, as well as between measurement dates, the effect of the train datasets was comparably small. Generally, within-trials models achieved better predictions (max. R^2^ = 0.27–0.81), but R^2^-values for the best across-trials models were lower only by 0.03–0.13. Within train and test datasets, measurement dates had a strong influence on model performance. While measurements during flowering and early milk ripeness were confirmed for within- and across-trials models, later dates were less useful for across-trials models. For most test sets, multi-date models revealed to improve predictions compared to individual-date models.

## 1. Introduction

Substantial work has been conducted in recent years with the aim of improving non-destructive, sensor-based grain yield (GY) prediction for plant breeding. Based on improved, less expensive and more user-friendly sensor platforms such as drones or UAV (unmanned aerial vehicles), better camera sensors with better spectral characteristics, improved vegetation indices or machine learning algorithms, GY prediction was improved [1,2,3,4,5,6]. Besides RGB-based sensing, multispectral sensing is recommended due to its higher sensitivity in the red edge and near infrared (NIR) spectrum [4,6].

Several studies have shown that the time of measurement, i.e., the growth stage during data collection, is crucial. Often measurements at milk ripeness were found to be most useful for winter wheat [3,7] and spring wheat [8,9,10]. In contrast, predictions were found to be weak during vegetative growth and to decrease during ear emergence and later grain filling [7,10,11], whereas other studies reported increasing correlations during grain-filling [12,13]. Thus, optimum time slots for UAV missions differ depending on location, genotypes, years and weather conditions. Consequently, the transfer of models between trials is not trivial.

Many of these previous studies focused on the application of the methods in individual trials or compared results from various trials. However, model transferability, i.e., training and independent application of models in other trials, remains crucial for actual prediction instead of a posteriori ‘postdiction’. A study on GY prediction in spring barley reported good transferability of models across years from spectral data collected during anthesis using partial least squares regression (PLSR) [14]. For wheat, R^2^-values for across-years GY predictions from milk ripeness measurements differed between three pairwise combined years from 0.16–0.50 [7]. Results were more influenced by the test set compared to the training set. However, substantial under- or overestimation was observed in both studies, indicating an offset between spectral data and GY in different trials. Spectral data was reported to improve genomic GY predictions within, but not consistently across environments [15].

A study on GY prediction in spring wheat [16] assessed the effect of different years and locations with combinations of data from different trials and independent testing on other trials. Independent GY prediction explained 83% variance based on a single training trial, whereas additional trials for training did not consistently improve predictions. Due to year-specific weather conditions, a tendency for improvements from year specific models was found. In addition, locations differed significantly in terms of prediction accuracies. Thus, the location with the highest soil quality and GY showed the lowest prediction accuracies. The authors pointed out limitations in model transfer due to differing growth conditions, weather conditions during spectral measurement and the influence of weeds on spectral signature. However, the study included differing nitrogen fertilization treatments [16], limiting the transfer to breeding applications.

In a study on GY prediction between three years for two rice cultivars and multiple nitrogen levels based on multispectral UAV-based data, correlations differed between years, cultivars and two sensors [17]. Depending on the year combinations, either a normalized red edge reflectance or a texture index was recommended. Likewise, models specific to years and locations performed slightly better than global models for forage yield estimation [18], indicating that similarity in the training and test conditions may be more important than the amount of training data.

Considering the limited studies on GY modelling for predicting genotypic differences in GY in different locations and years, this analysis aims to evaluate the dataset effect for GY prediction in winter wheat breeding yards based on data from three years and two locations. It extends the evaluation of within-trials models from two locations and two years [19]. This study recommended a red edge index amongst different RGB and multispectral indices and measurements around anthesis and early milk ripeness. Amongst machine learning algorithms, comparable small differences in accuracies were found. The combination of measurement dates generally improved predictions. In the present study, across-trials models are compared to within-trials models. ‘Trial’ refers to one field trial within a particular year at one particular location. Since models need to achieve sufficient prediction accuracies within particular trials, testing is only conducted within trials. Moreover, based on the results in the analysis from Prey et al. (2022) [19], where GY predictions differed substantially between locations and years, we expect that the combination of data from multiple trials for model training will not directly improve GY predictions, since models would be fitted to the inter-trial variation as opposed to the intra-trial variation. We expect that the use of models trained on trials most similar to the trial used for application will be more expedient. Therefore, either year-specific models or location-specific models might perform better. Different combinations of training and test datasets are evaluated, including (i) models ‘within years within locations’ (WYWL), (ii) ‘within years across locations’ (WYAL), (iii) ‘across years within locations’ (AYWL) and (iv) ‘across years across locations’ (AYAL).

## 2. Materials and Methods

### 2.1. Field Trials

Field trials were conducted using a plant breeder in two locations in southeast Germany, near Herzogenaurach (“HZ”; approximately 10.86 E, 49.55 N) and in eastern Germany, near Morgenrot (“MR”; approximately 11.21 E, 51.78 N), each in the growing seasons 2019/20, 2020/21 and 2021/22. The six year*location combinations are denoted as HZ_20, MR_20, HZ_21, MR_21, HZ_22, and MR_22 for locations HZ and MR in the first, second and third year, respectively. Both locations are situated in the Köppen–Geiger climate zone Dfb (“Warm-summer humid continental climate”). In comparison to HZ, where soil texture was quite variable and included sandy, silty and loamy soil (Stagnosol) over heterogeneous terrain, MR trials were characterized by increased water retention capacity with more homogeneous soil (Tschernosem) and flat terrain.

Both trials were impacted by pronounced drought in 2020 and 2022, while growing conditions were more favorable in 2021. Thus, 2020 was characterized by relatively dry and hot conditions during April and May, the months of main vegetative growth, whereas the second year was colder and more humid (Figure S1). In 2022, the April temperature in both locations was between that recorded in 2020 and 2021, respectively. While the April precipitation in HZ was clearly higher in 2022 than in previous years, it was below 25 mm in MR. In both locations, precipitation during May and June was even lower and air temperature higher in 2022 than in 2020.

Within years, both trials comprised identical germplasm, consisting of pre-selected material without extreme genotypes in terms of morphology and phenology, F5 generation and older as well as double haploid lines. To account for the heterogeneous soil, every fifth trial row was sown with one of two standard (‘references’) cultivars, whereas other genotypes were sown without replication.

Sowing dates were mostly in the second half of October and harvest dates in the last and first week of July and August, respectively. Preceding crops were rapeseed, sugar beet and rapeseed for the HZ trials, and rapeseed for the MR trials for the trials in 2020, 2021 and 2022, respectively. Plots were kept weed-free and fertilized in compliance with local standards. In contrast to MR, no fungicide was applied in the HZ trials.

Plots were harvested using a combine harvester for determining grain yield (GY) at kernel moisture of 14%. A fraction of the plots were neglected for harvest, notably based on pathogen scores, weak biomass growth or plot damage due to lodging. GY was available for 4423 plots from a total of 4930 plots in MR_20, for 4349 from a total of 4923 plots in HZ_20, for 2787 plots from a total of 3636 plots in MR_21, for 2711 plots from a total of 3588 plots in HZ_21, for 1785 plots from a total of 2766 plots in MR_22 and for 1869 plots from a total of 2766 plots in HZ_22 (Table S1).

### 2.2. Sensor Measurements and Data Preprocessing

Multispectral (MS) drone-based data was acquired during all major growth stages throughout the growing season, on 8, 9, 7, 6, 8 and 13 dates in the MR_20, HZ_20, MR_21, HZ_21, MR_22 and HZ_22 trials, respectively (Table 1). A Tetracam µMCA (Tetracam Inc.; Chatsworth, CA, USA) was used in MR_20, HZ_20, HZ_21 and HZ_22 and a Tetracam MCAW in MR_21 and MR_22, with the exception of June 9 in MR_22, when the Tetracam µMCA was also used. Both cameras are equipped with the same snapshot sensors, thus, capturing the entire picture at the same time. The focal length of the optics of both cameras was 9.6 mm. The resolution of the band-specific CMOS sensors was 1.3 MP for both cameras, resulting in individual images of 1024 × 1280 pixel extent. Radiometric resolution was 8 and 16 bit depth for the µMCA and MCAW data, respectively. The cameras were carried by a DJI M600 or a XR6 (AIR6 Systems GmbH, Klagenfurt am Wörthersee, Austria). The cameras comprised six bands each, including a red edge band and a near infrared band, which were used in the present study. The forward and sideward image overlap were targeted to be at minimum 80% and 60% in MR and 80% and 75% in HZ location, respectively. Flight height was about 60–80 m and flight speed typically 4–8 ms^−1^. The resulting pixel size of the orthomosaics was 3–6 cm. However, additional resampling of the image data showed that the applied methods are robust with respect to the effect of the pixel resolution (not shown). 

PixelWrench for Macaw or PixelWrench 2 was used to preprocess the multispectral cameras’ raw data (Tetracam Inc.; Chatsworth, CA, USA). The data was calibrated using values from a 22% grey reflectance standard that was recorded immediately after takeoff or before the UAV touched down. Spatial co-registration was carried out for each band to account for the spatial offset. Reflectance was calculated by dividing the up-welling radiation measured in each band by the down-welling radiation measured simultaneously using the incident light sensor. Images were processed for image alignment and the generation of a sparse and dense point cloud, based on which the orthomosaics were generated using the software Agisoft Metashape Professional Edition (versions 1.5–1.8; Agisoft LLC., St. Petersburg, Russia). Based on ground control points (GCP) taken with an RTK-enabled GNSS-device with an accuracy of 1.5 cm at nine positions in each of the trials, the data was georeferenced to an accuracy of 2–3 cm. Based on the GNSS-guided sowing of the trials, parcel boundary polygons were created using the software MiniGIS (versions 2.11–2.13; geo-konzept GmbH, Adelschlag, Germany).

### 2.3. Postprocessing of Spectral Data and Grain Yield Modelling

The methodology is based on that described in detail in Prey et al. (2022) [19]. Based on that study [19], which identified the normalized difference red edge index (*NDRE*1) as overall best vegetation index for GY estimation among several tested multispectral and RGB bands and vegetation indices, this study focuses on this spectral index. The index was calculated as

$$NDRE1=\frac{\rho_{NIR1}-\rho_{RE1}}{\rho_{NIR1}+\rho_{RE1}}$$

With $\rho_{NIR1}$ and $\rho_{RE1}$ denoting reflectance in the NIR and red edge bands, centered at 780 nm and 700 nm, respectively. The full width at half maximum (FWHM) band width was 10 nm. The vegetation index was calculated on the pixel level using the input raster layer in a custom-made analysis pipeline developed in Python [20]. The raster data was extracted on the plot level using “Grid Statistics for Polygons” in SAGA [21]. All quantiles were calculated at intervals of 5%, including minimum, median and maximum, as well as the standard deviation and mean values. Plot boundaries were buffered inside by 20 cm in order to exclude the boundary pixels.

The data was analyzed in R [22] using an automated processing pipeline based on the caret package [23]. The partial least squares regression (PLSR) was chosen based on its overall good performance and its calculation efficiency in the previous analysis within trials [19]. The *NDRE*1 input data was centered to mean 0 and scaled to standard deviation 1. By default, all statistical variables of the *NDRE*1 at the plot level were included as predictors, thus, including 23 predictors per measurement day. All data was used as aggregated on the plot level. Outliers were automatically detected and filtered based on local outlier factor [24] identified from the Mahalanobis distance following principle component analysis using the R-package “bigutilsr” [25,26].

PLSR parameters were tuned using 10-cross validation. The optimum number of latent variables was determined based on the minimum root mean squared error (RMSE) in the cross validation. The predictions on the test set data were compared to the measured yield. The comparison focuses on the R^2^-values since GY prediction in phenotyping requires relative discrimination of the data, but, unlike the situation for precision farming [27], can generally tolerate an offset in the predictions [28]. Values for mean absolute error (MAE), root mean squared error (RMSE), relative RMSE (RRMSE_mean), bias and offset are reported in the supplementary material.

### 2.4. Dataset Combinations for Grain Yield Modelling

**Figure 1:**
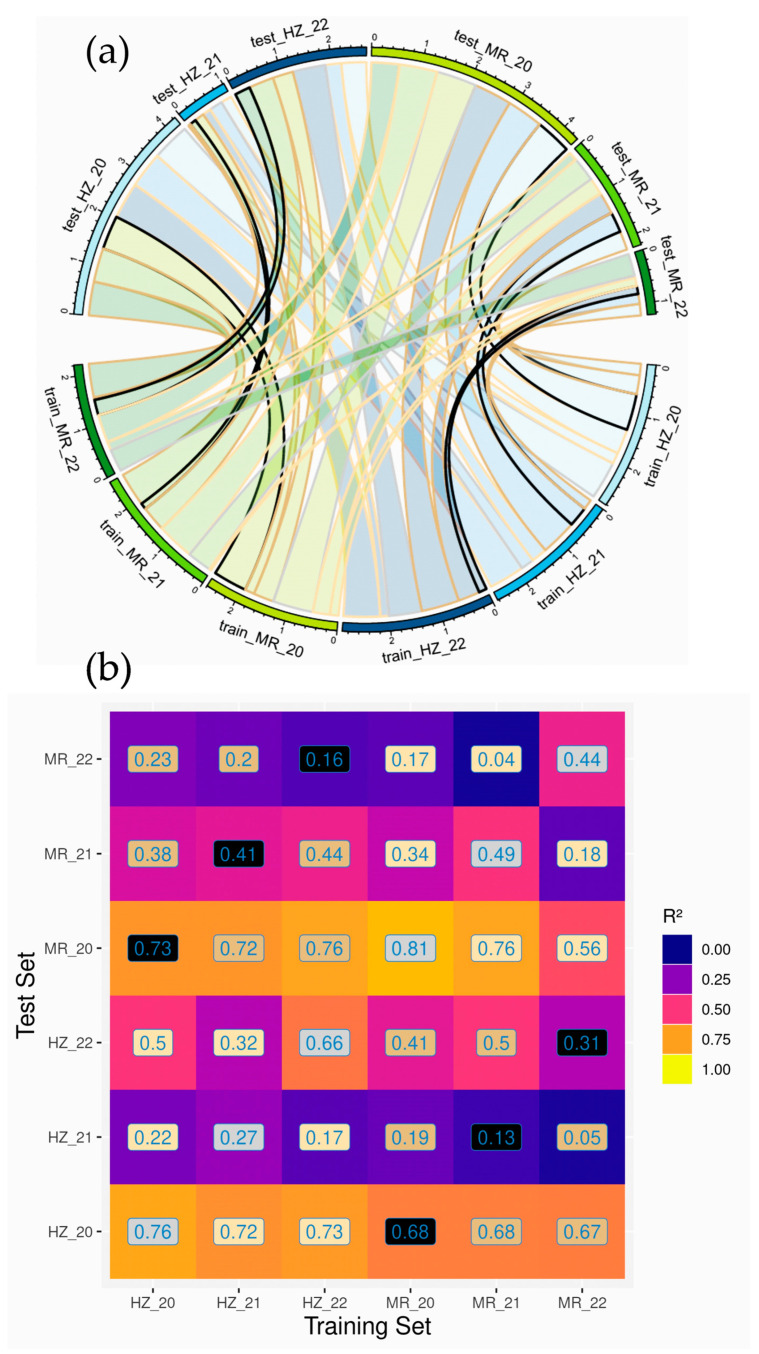
Visualization of coefficients of determination (R^2^) for the train*test set combinations for the ‘full models’ based on all matched measurement days (‘all times’). Green (MR) and blue (HZ) colors in (**a**) denote the locations, color intensity the three years, respectively. The width of the connecting bars represents the R^2^-levels and the outer, circular bars the cumulative R^2^-values in the test evaluation. Boundary colors in (**a**) and label background colors in (**b**) denote the dataset combinations (W: within, A: across, Y: year and L: location): WYWL (grey), WYAL (black), AYWL (light beige) and AYAL (dark beige).

Models for GY estimation were trained and evaluated with respect to the individual trials used for training and testing, referred to as ‘trial combination types’, i.e., within or across years (WY and AY, respectively), and within or across locations (WL and AL, respectively). The combinations of both factors result in four model types ‘within years within locations’ (WYWL), ‘within years across locations’ (WYAL), ‘across years within locations’ (AYWL) as well as ‘across years across locations’ (AYAL). Thus, for each of the six trials used as a test set, six datasets were used for model training, resulting in 36 dataset combinations as visualized in the connecting bars in Figure 1a. In the case of across-trials models, all plots of the test trials were used as a test set.

The WYWL models were reported in [19] for the trials in 2020 and 2021 and evaluated in the same way for the MR_22 and HZ_22 trials, based on an independent test set using 20% of the data based on random data splitting. For modelling across trials, dates were matched between trials based on the least temporal differences as calculated from the day of the year (DOY), assuming that the measurement dates, i.e., for the training and the test data should be the most similar. Thus, for each training date, the testing date with the lowest difference in terms DOY was used. Dates were translated to approximate growth stages according to the Zadok’s scale, e.g., 25_30, where 25 and 30 denote the growth stage for the train data and 30 the growth stage for the test data, respectively.

As for the WYWL analysis in Prey et al. (2022), besides individual-date models, multi-date models were tested, either using the complete seasonal data (“all times”) or by adding incrementally the data from the next measurement date (date increments 1 to *i*, with *i* being number of measurement dates − 2). Thus, for example, “date_increment_1” comprises the data from the first and second date.

Results reported for WYWL models for the years 2020 and 2021 correspond to those reported in [19] for the chosen spectral data and PLSR algorithm, but slightly differ for MR_20 due to an additional date: May 19.

## 3. Results

### 3.1. The Effect of Train and Test Datasets

Based on the full models with all matched dates, the train*test set combination types are compared for the 36 pairwise trial combinations (Figure 1). Within all test sets, the WYWL models (grey color), trained on independent data of the same trial, achieved best predictions, yet on different levels. Coefficients of determination (R^2^-values) were highest in MR _20 (R^2^ = 0.81) and HZ_20 (R^2^ = 0.76), followed by HZ_22 (R^2^ = 0.66), MR_21 (R^2^ = 0.49), MR_22 (R^2^ = 0.44) and HZ_21 (R^2^ = 0.27; Figure 1b). Thus, the effect of year of the test set, with high values in 2020 but low values in 2021 in both locations, was dominant compared to the effect of location, with higher values in MR in 2021, but lower values in 2022 compared to HZ.

For a given test set, the effect of the training set was comparably low, as is visible from the similar cumulative R^2^-values in the lower half of the figure (Figure 1a). In contrast, as in the within-trials models (WYWL), results in different test sets differed substantially.

### 3.2. The Effect of Across-Trials Model Types

**Figure 2:**
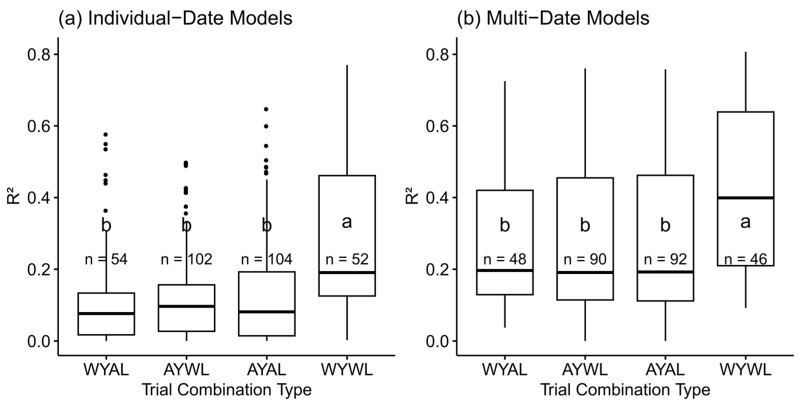
Comparison of coefficients of determination (R^2^) for the train*test set trial combinations. W: within, A: across, Y: year and L: location. (**a**): Comparison of individual-date models and (**b**) Comparison of multi-date models. Letters denote groups according to Tukey’s post hoc test, numbers the number of compared models. See Figure S3 for further metrics.

Across-trials models were described by two factors ‘year’ and ‘location’, as well as their combinations. The variation of the R^2^-values was compared both for the model groups ‘individual-date’ (Figure 2a) and ‘multi-date’ (Figure 2b). In both groups, WYWL models achieved significantly (*p* < 0.05%) better predictions than the other model types. However, R^2^-values of the across-trials model types (WYAL, AYWL and AYAL) did not differ significantly amongst each other. These results are complementary with lower RMSE, RRMSE_Mean and MAE values from the WYWL models—both in individual and multi-date models (Figure S3). In contrast, bias and offset were not significantly different between the across-trials and WYWL models. However, bias was always close to zero in WYWL models, but showed considerable positive and negative variation between across-trials models (Figure S3). Unlike for R^2^, RMSE and RRMSE_Mean were lower in WYAL than in AYAL multi-date models. Likewise, MAE was lower in WYAL and AYWL than in AYAL models, but still higher than in WYWL multi-date models.

### 3.3. The Effect of Measurement Time for Individual-Date Models

**Figure 3:**
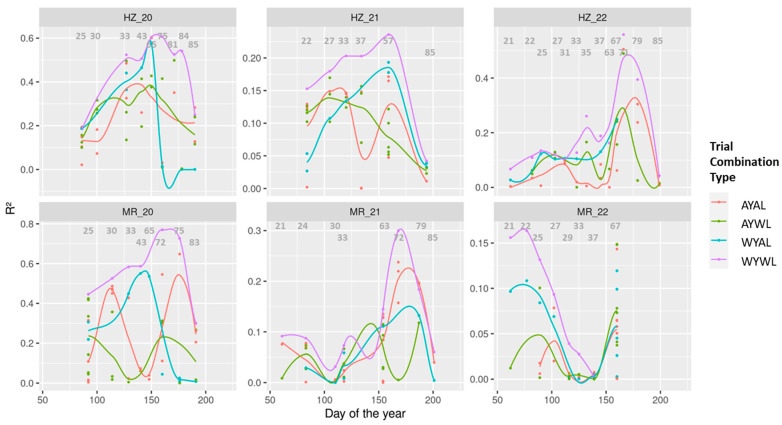
Comparison of coefficients of determination (R^2^) for the individual-date models by test set trial (sub-figures) and by trial combination type (colors). W: within, A: across, Y: year and L: location. Colored lines are fitted using local polynomial regressions. Grey numbers indicate approximate growth stage numbers. See Figure 4 for comparisons with multi-date models.

In all trials except that of MR_22, R^2^-values in individual-date models increased over time in the within-trials (WYWL) models (Figure 3; violet color), peaked around anthesis and early milk ripeness and steadily decreased thereafter. In general, this temporal pattern was similar for the across-trials models.

For each test set, the WYAL models correspond to only one train set per time slot. In HZ_20, WYAL models peaked at a similar R^2^-level as WYWL models but decreased sharply after anthesis. This pattern was similar in MR_20, yet with a higher level in WYWL models. In 2021, the temporal pattern of WYAL models followed that of WYWL models, but on a clearly lower level with maximum R^2^-values below 0.20. In MR_22, data was sparse during the grain-filling phase. R^2^-values were low (R^2^ < 0.20) during the vegetative phase and still at anthesis. In HZ_22, WYWL and WYAL models followed a similar pattern, but WYAL models were mostly missing post anthesis due to missing data in MR_22.

For each test set, two AYAL and AYWL models were available per measurement date, respectively. In HZ_20, results for both model types had a similar seasonal shape with lower values before, but higher values after anthesis, compared to the WYAL model. The results were less consistent in MR_20, with low predictions from some models around anthesis. In HZ_21, predictions from AYAL and AYWL models were mostly lower than from the WYAL and especially WYWL model. In HZ_22 and MR_22, the seasonal pattern of both model types was similar to that of the WYWL model, but again clearly on a lower level.

### 3.4. The Value of Multi-Date Models

Multi-date models include data from two up to all measurement dates, depending on the number of matched dates between train and test datasets. Dates were incrementally added in temporal order. Scatterplots in Figure S2 depict exemplary WYAL model predictions for individual-date models (first row in (a), (b) and (c), respectively, and multi-date models, second row, respectively). The best R^2^-values from all multi-date models are compared to those from individual-date models from all trial combinations (Table 2). In all except two train*test set combinations, the best predictions from multi-date models were higher than those from individual-date models (Table 2). The advantage of best multi-date models did not show particular patterns but tended to be relatively stronger in some of the across-trials combinations. In 2021, irrespective of the training trial, improvements were pronounced in MR_21, but marginal in HZ_21.

**Figure 4:**
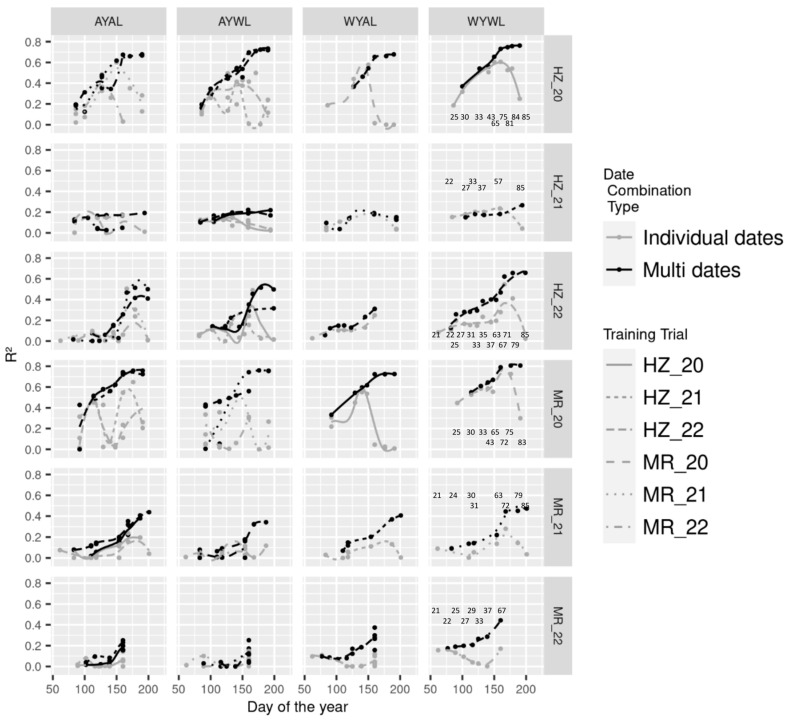
Comparison of coefficients of determination (R^2^) by trial combination types (columns), date combination types (grey and black lines), test trials (rows) and training trials (line types). W: within, A: across, Y: year and L: location. Lines are fitted using local polynomial regressions. Results for WYWL correspond to those in Figure 3. For the incremental multi-date model (black), the R^2^-value is positioned at the measurement date of the last included date. Thus, multi-date models include dates in the temporal order of measurement. The full model is positioned at the rightmost position, respectively. See Figure S4 for comparisons of further evaluation metrics. Numbers within WYWL figures indicate approximate growth stages.

Figure 4 depicts differences in R^2^-values of multi-date models in comparison to those from individual-date models by measurement time. With only a few exceptions, multi-date models, based on included data until a particular measurement date, improved predictions from individual-date models at this date. While improvements were marginal for many earlier dates during vegetative growth, they were more pronounced during later growth stages, in particular for the MR_20 and HZ_20 test sets. The improvements from multi-date models over individual-date models at best individual measurement dates differed between train*test set combinations (Figure 4, Table 2). Thus, improvements were found in WYWL models in HZ_20 and HZ_22, but hardly in the across-trial models WYAL, AYWL and AYAL. In contrast, in MR_20, improvements were neglectable in WYWL but more pronounced in AYWL and AYAL models.

## 4. Discussion

Spectral GY models can be trained and applied based on different data sources. However, when training and test data are generated from the same field trials, test sets do not reflect real-world prediction application cases, where no training data would be available from trials in which models should be applied. This study, therefore, assessed the application of models trained from the data of individual trials to trials in other years and locations.

### 4.1. The Influence of Combinations of Training and Test Datasets

As in the previous analysis within four of the included trials [19], prediction accuracies differed strongly between trials. With respect to the trial combinations, the dominant factor was (i) the data available for applying the models in the test set rather than (ii) the trial the training data was generated from. Thus, neither a location-specific nor a year-specific strategy was significantly better in the relative GY discrimination, as expressed using the coefficient of determination (R^2^), than in ‘across years, across location’ (AYAL) models. In spite of mostly similar year-specific weather conditions (Figure S1), growing conditions differed significantly between both locations, due to better soil quality and—in contrast to HZ—fungicide treatment in MR. Likewise, weather conditions differed strongly between years within locations. Thus, GY differed significantly between both locations within all years as well as between all years within locations, but not between HZ_20 and MR_22 (Table S1). This may explain that in spite of strong R^2^-variation within the three across-trials strategies (Figure 2), none of them was superior. The dominant influence of the test set data is in line with across-years GY prediction from milk ripeness measurements in one location for winter wheat [7] as well as the significant effect of both location and year for GY prediction in spring wheat [16]. While predictions in this study [16] mostly profited from data from the same year in train and test sets, in some cases across-years models performed better, similar to the present results.

In contrast to the results for R^2^, the mean absolute error (MAE), root mean squared error (RMSE) and relative RMSE were lower in multi-date models for ‘within years, across location’ models than in ‘across years, across location’ (AYAL) models (Figure S3). This indicates that the differences in absolute GY levels, which were mostly significant between locations and years, resulted in higher errors in the AYAL models.

The weak predictions in both locations in 2021 are likely to be related to the moister conditions in this year, which resulted in increased vegetative biomass, and therefore, likely saturation of the spectral data [12]. Moreover, unlike the situation in the other years, fungal diseases were relevant, which were reported to counteract spectral predictions over time [9]. In contrast, the weak prediction in MR_22 is likely influenced by the lack of dates during grain filling—the phase when useful predictions were observed in HZ the same year.

### 4.2. The Effect of Measurement Dates

This study confirms the seasonal pattern for GY prediction with generally weak predictions before flowering but relatively better predictions during flowering and milk ripeness for the ‘within years within locations’ (WYWL) models (see Figure 4) [19], which is confirmed by a number of previous studies [2,3,7,8,9]. During this stage, vegetative growth is mostly terminated, the yield components ear number and kernel density are mostly determined [29] and the spectral signal is still little distorted by shifted senescence [30]. Across-trials models are sensitive to the ‘translation’ of measurement dates between training and test data. As a simplified approach, which requires no additional information, dates were matched based on the least temporal difference and translated to growth stages. While approximate growth stage information was available, its determination is error-prone and not feasible for thousands of trial plots. In addition, it is likely that growth stage as scored on the kernels does not reflect the canopy appearance notably of leaves, which, however, is dominant for the spectral signal [31]. Thus, the date translation was affected by trial-specific shifts in phenological development and by differing measurement frequencies between trials (Table 1). Therefore, not all dates in the test datasets could be used or important dates were missing, notably in MR_22 post anthesis. Moreover, imperfect date translation may explain that in spite of good predictions of the WYWL models within both trials during milk and early dough ripeness in 2020, these stages were not useful any more in the across-trials models and exceptionally high RMSE values were observed (Figure S4).

In addition, although the spectral data was captured only under suitable measurement conditions, potential influences from differing illumination intensities, wind speed, flight height, temperature and possible effects of background dark current should be addressed in more detail for optimizing both the merging of data from different measurement dates and the date translation.

### 4.3. Multi-Date Models

As GY is influenced over time by growing conditions in all growth stages, the incremental combination of measurement dates generally increased prediction accuracies and decreased RMSE, RRMSE_Mean, MAE and Offset values (Figure S4). These improvements were steady and mostly increased with more included dates, at least until anthesis, indicating that information from multiple growth stages was important. This is in line with previous studies reporting mostly moderate improvements from multiple measurement dates [12,32,33,34]. However, a saturation was observed as soon as the best individual dates were included, especially in the WYWL models (Figure 4). Thus, the predictions did not further profit from measurements during late dough ripeness. Still, multi-date models showed the advantage of temporal stability with rarely decreasing predictions during late growth stages (Figure 4).

Improvements were not consistently stronger for cases of weaker predictions from individual-date models. This indicates that the test set limitation from unsuited measurement dates or unfavorable conditions, as visible especially for HZ_22 (Table 2), could not be compensated for by date combinations. In addition, the clearly stronger relative improvements from multi-date models in MR_21—irrespective of the training set—indicate that besides the dominant effect of the test set for the maximum prediction potential, the improvement from multi-date modelling was also dominated by the test rather than the training set.

### 4.4. Limitations and Outlook

The results indicate that GY prediction shows good potential under favorable conditions in HZ_20, MR_20 and HZ_22, whereas distinct limitations became evident in the other trials and in multi-date models irrespective of the trials used for training. Given the known differences in soil and weather conditions between trials, and contradicting results from ‘global models’ in the literature, we deliberately have not included global models in this study. Moreover, only one spectral trait, *NDRE*1, and one algorithm, PLSR, were used. More spectral traits should be tested, as well as more machine learning algorithms. Thus, random forest was recommended in a number of studies [35,36,37], notably for its ability to handle heterogeneous data. This aspect might be more relevant in across-trials compared to within-trials models, in which prediction accuracies had been similar, but training time significantly higher [19]. In addition, the translation, i.e., matching of dates from different trials, should be further optimized. Thus, possible phenological shifts of the canopy in spite of similar growth stage should be addressed. Furthermore, besides the incremental data combinations, further date combinations should be tested for alleviating the effort of additional measurements. Moreover, two camera models were used in this study, which, however, use the same sensor. A previous simulation analysis for comparing multiple satellite sensors found very close relationships of R^2^ = 0.99 between sensors for the normalized difference vegetation index [38], thus, indicating sufficient consistency of the data of the included cameras. Since some trial combinations with different cameras showed better results than other trial combinations with the same cameras, the effect of different cameras appears to have been neglectable but should be further addressed.

## 5. Conclusions

This study extended GY prediction within trials to three across-trial cases of predictions across years and locations. In addition, individual-date and multi-date models were compared. While the feasibility of GY prediction was also confirmed for across-trials cases, prediction accuracies generally slightly decreased. The influence of the different trial-specific training datasets was relatively low; thus, neither a location-specific nor a year-specific strategy performed better. In contrast, the test data as influenced by the year- and location-specific growing conditions, was most influential for the prediction accuracies. In addition, pronounced differences between measurement dates were confirmed, with generally better predictions around anthesis and early milk ripeness. However, some growth stages, especially post anthesis, were less reliable compared to within-trials models. Both within and across trials, date combinations were promising for improved predictions if useful individual dates were included.

## Figures and Tables

**Table 1:** Overview on UAV measurements: Dates (year–month–day) with approximate average growth stages (Zadok’s scale) for the two locations (MR and HZ) and three years. * 27 May 2022 was not included in the previous analysis [19], due to missing RGB data.

Trial	Date	Growth Stage	Trial	Date	Growth Stage
MR_20	1 April 2020	25	HZ_20	26 March 2020	25
	23 April 2020	30		9 April 2020	30
	8 May 2020	33		6 May 2020	33
	19 May 2020	43		20 May 2020	43
	27 May 2020 *	65		29 May 2020	65
	8 June 2020	72		8 June 2020	75
	24 June 2020	75		19 June 2020	81
	9 July 2020	83		26 June 2020	84
				26 July 2020	85
MR_21	24 March 2021	24	HZ_21	25 March 2021	22
	20 April 2021	30		15 April 2021	27
	28 April 2021	31		30 April 2021	33
	3 June 2021	63		14 May 2021	37
	17 June 2021	72		8 June 2021	57
	6 July 2021	79		13 July 2021	85
	20 July 2021	85			
MR_22	3 March 2022	21	HZ_22	3 March 2022	21
	18 March 2022	22		23 March 2022	22
	30 March 2022	25		31 March 2022	25
	12 April 2022	27		13 April 2022	27
	26 April 2022	29		22 April 2022	31
	5 May 2022	33		3 May 2022	33
	19 May 2022	37		12 May 2022	35
	9 June 2022	67		25 May 2022	37
				2 June 2022	63
				9 June 2022	67
				15 June 2022	71
				28 June 2022	79
				18 July 2022	85

**Table 2:** Comparison of individual-date models and multi-date models by maximum prediction accuracy (R^2^-values) by trial combination type (W: within, A: across, Y: year and L: location) as well as training and test set combinations. R^2^-difference was calculated as the difference between multi-date and individual-date models.

Train Set	Test Set	Individual Dates (R^2^)	Multi Dates (R^2^)	R^2^ Difference
		WYWL		
HZ_20	HZ_20	0.61	0.76	0.16
HZ_21	HZ_21	0.24	0.27	0.03
HZ_22	HZ_22	0.55	0.66	0.11
MR_20	MR_20	0.77	0.81	0.04
MR_21	MR_21	0.30	0.49	0.19
MR_22	MR_22	0.17	0.44	0.27
		WYAL		
MR_20	HZ_20	0.58	0.68	0.10
MR_21	HZ_21	0.19	0.19	0.00
MR_22	HZ_22	0.25	0.31	0.06
HZ_20	MR_20	0.55	0.73	0.17
HZ_21	MR_21	0.13	0.41	0.27
HZ_22	MR_22	0.12	0.37	0.25
		AYWL		
HZ_21	HZ_20	0.41	0.72	0.30
HZ_22	HZ_20	0.50	0.73	0.24
HZ_20	HZ_21	0.15	0.22	0.07
HZ_22	HZ_21	0.17	0.22	0.05
HZ_20	HZ_22	0.49	0.52	0.03
HZ_21	HZ_22	0.17	0.32	0.15
MR_21	MR_20	0.36	0.76	0.40
MR_22	MR_20	0.42	0.56	0.14
MR_20	MR_21	0.12	0.34	0.22
MR_22	MR_21	0.09	0.18	0.08
MR_20	MR_22	0.08	0.17	0.09
MR_21	MR_22	0.15	0.25	0.10
		AYAL		
MR_21	HZ_20	0.60	0.68	0.08
MR_22	HZ_20	0.49	0.67	0.19
MR_20	HZ_21	0.16	0.19	0.03
MR_22	HZ_21	0.17	0.14	−0.03
MR_20	HZ_22	0.24	0.42	0.18
MR_21	HZ_22	0.51	0.51	0.01
HZ_21	MR_20	0.55	0.72	0.18
HZ_22	MR_20	0.65	0.76	0.11
HZ_20	MR_21	0.22	0.38	0.16
HZ_22	MR_21	0.24	0.44	0.20
HZ_20	MR_22	0.14	0.23	0.09
HZ_21	MR_22	0.08	0.25	0.17

## Data Availability

Data is available from the authors upon reasonable request.

## Supplementary Materials

The following supporting information can be downloaded at: https://www.mdpi.com/article/10.3390/s23084177/s1. Figure S1: Weather conditions in the six location*trial combinations. Figure S2: Scatterplots of the WYAL predictions between trials within years. Figure S3: Comparison of model evaluation metrics for model testing by trial combination type for individual-date models (left) and multi-date models (right). Figure S4: Comparison of model evaluation metrics by trial combination types (columns), date combination types (line colors), test trial (rows) and train trial (line types). Table S1: Descriptive statistics of grain yield by field trial.
