# Supplementary material for: The Transferability of Spectral Grain Yield Prediction in Wheat Breeding across Years and Trial Locations
Source: Sensors (Basel). 2023 Apr 21;23(8):4177. doi: 10.3390/s23084177 (PMC10145428; doi:10.3390/s23084177)
Supplement: Supplementary file 1 [file sensors-23-04177-s001.zip › sensors-2272128-supplementary.pdf]

sensors

MDPI

Supplementary Materials

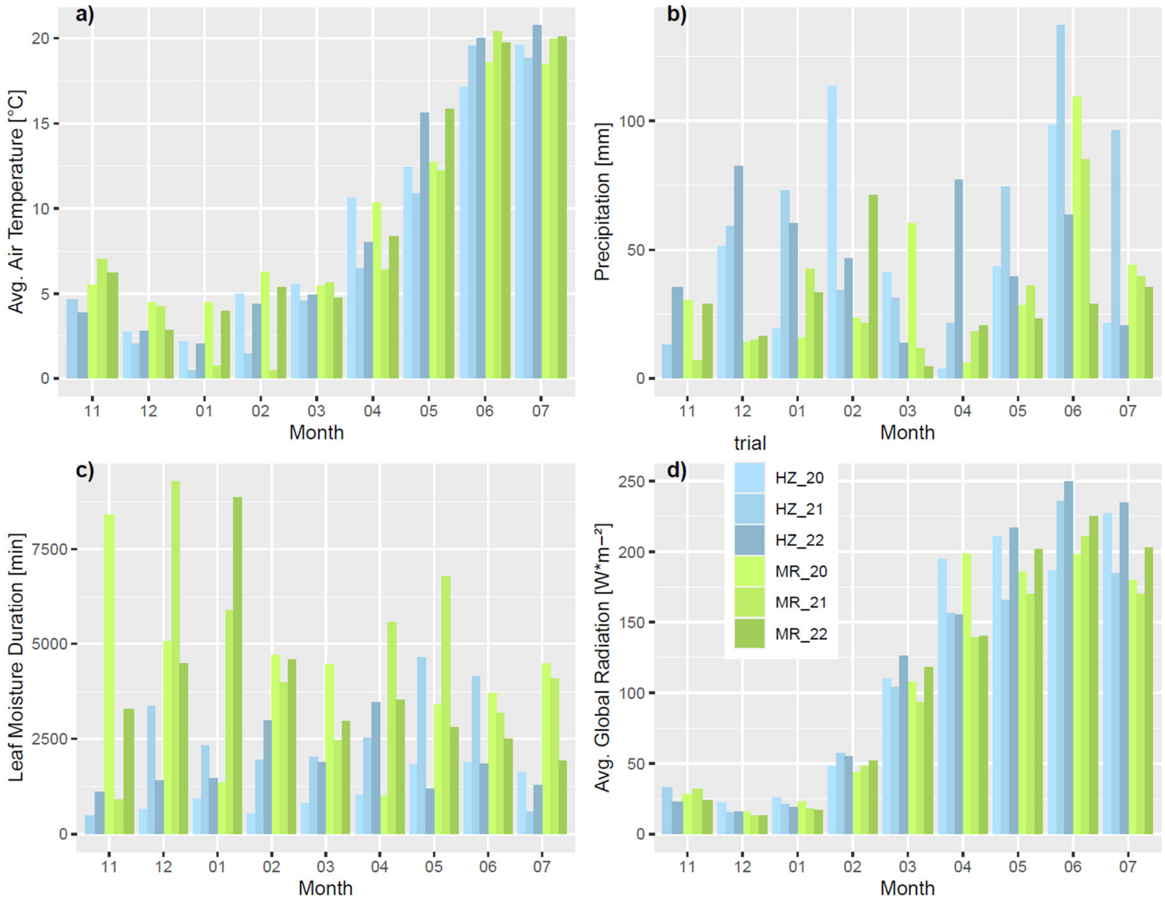

Figure S1. Weather conditions in the six location*trial combinations.

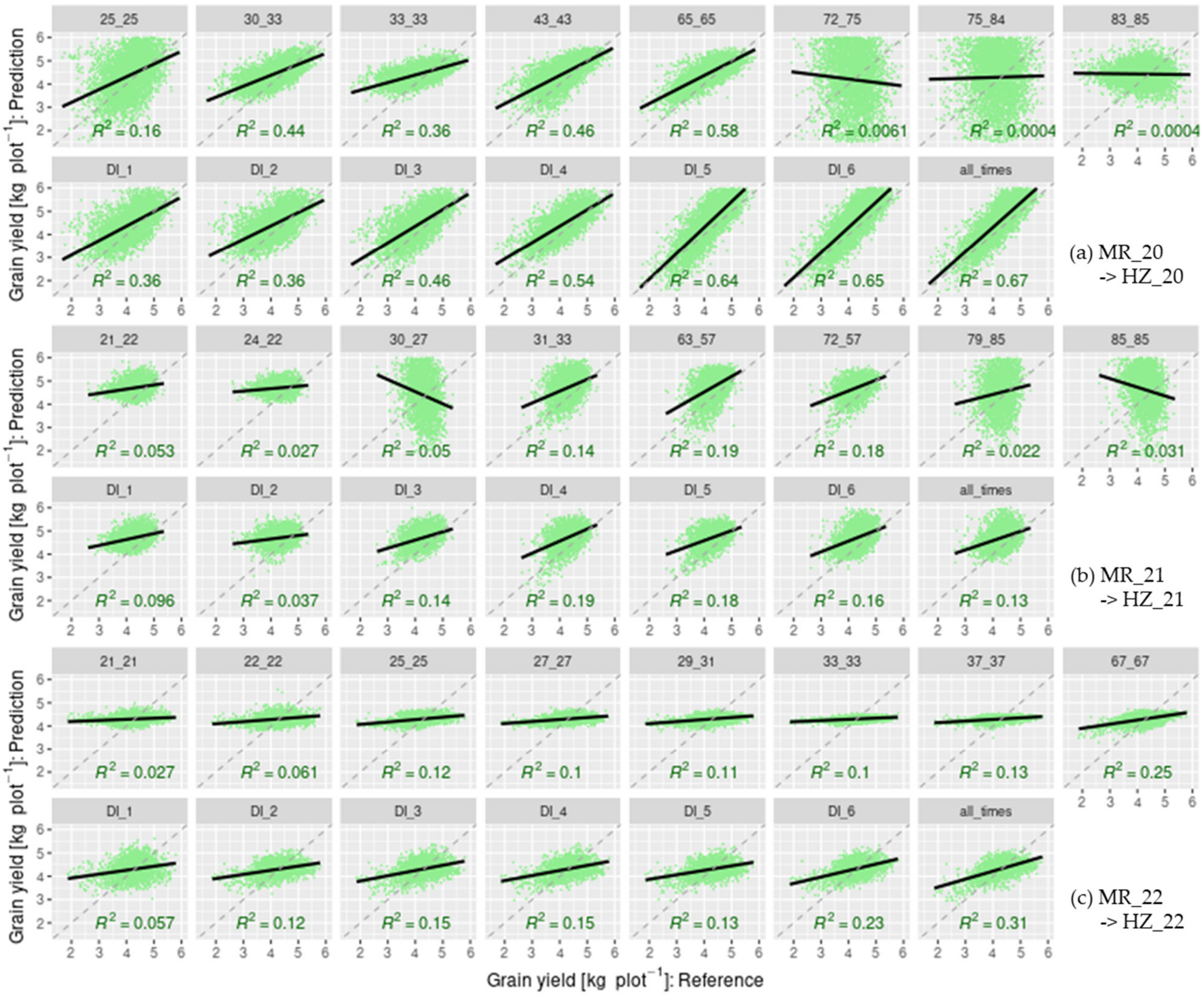

Figure S2. Scatterplots of the WYAL predictions between trials within years: Example of training in MR and testing HZ in 2020 (**a**), 2021 (**b**) and 2022 (**c**). 'DI': date increment, XX_YY denotes the growth stages for data used for training (XX) and testing (YY), respectively.

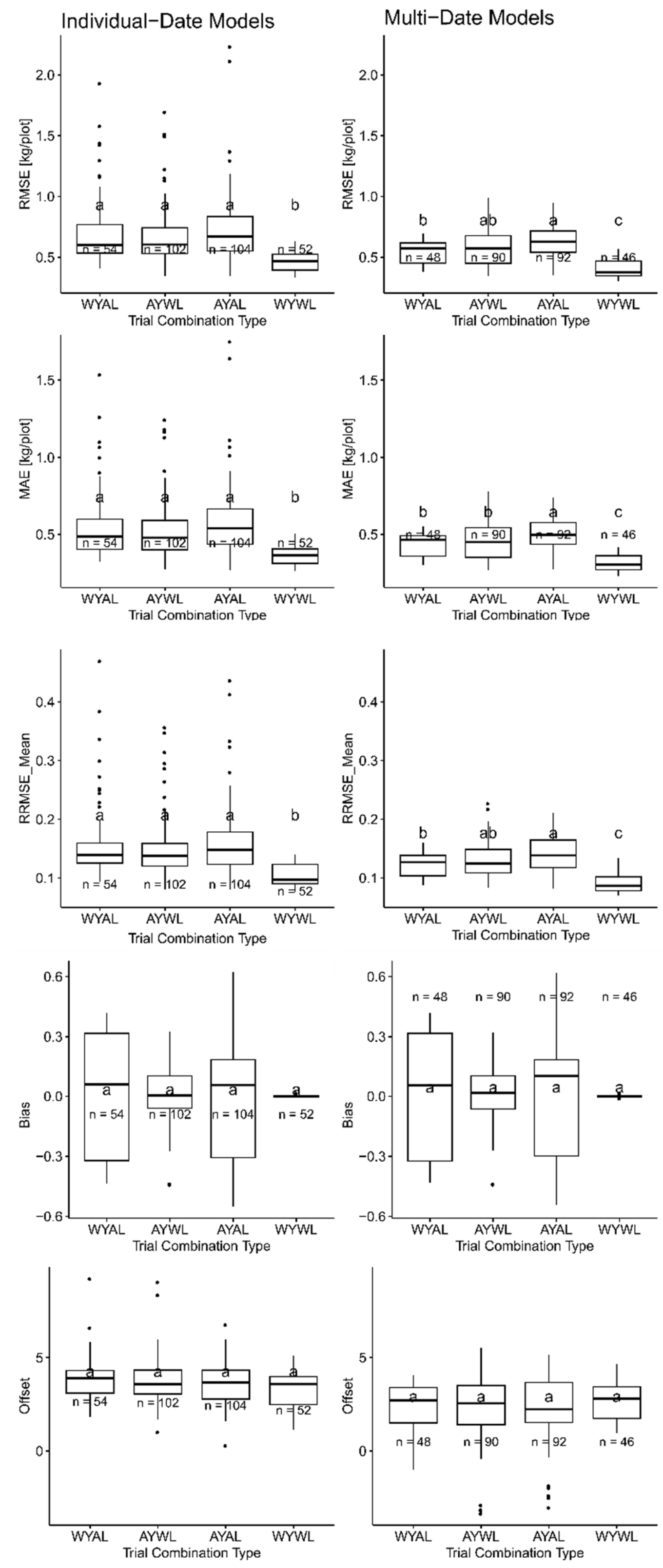

Figure S3. Comparison of model evalutation metrics for model testing by trial combination type for individual-date models (left) and multi-date models (right). RRMSE_mean was calculated by dividing RMSE by the mean GY and offset as the intercept from the relationship between predicted and measured GY. W: within, A: across, Y: year, L: location.

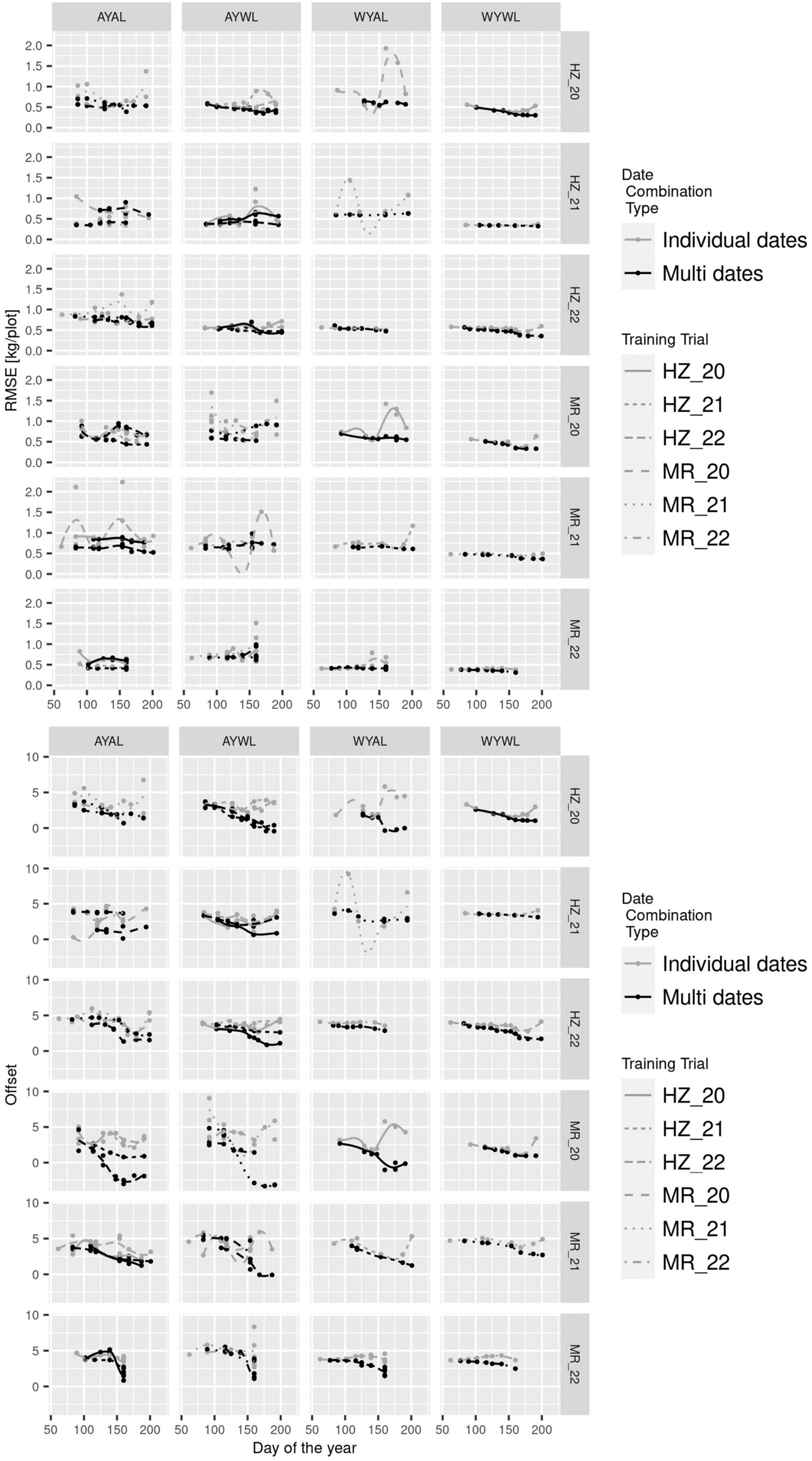
AYAL
AYWL
WYAL
WYWL
HZ_20
HZ_21
HZ_22
MR_20
MR_21
MR_22
RMSE [kg/plot]
Offset
Day of the year
Date Combination Type
Individual dates
Multi dates
Training Trial
HZ_20
HZ_21
HZ_22
MR_20
MR_21
MR_22

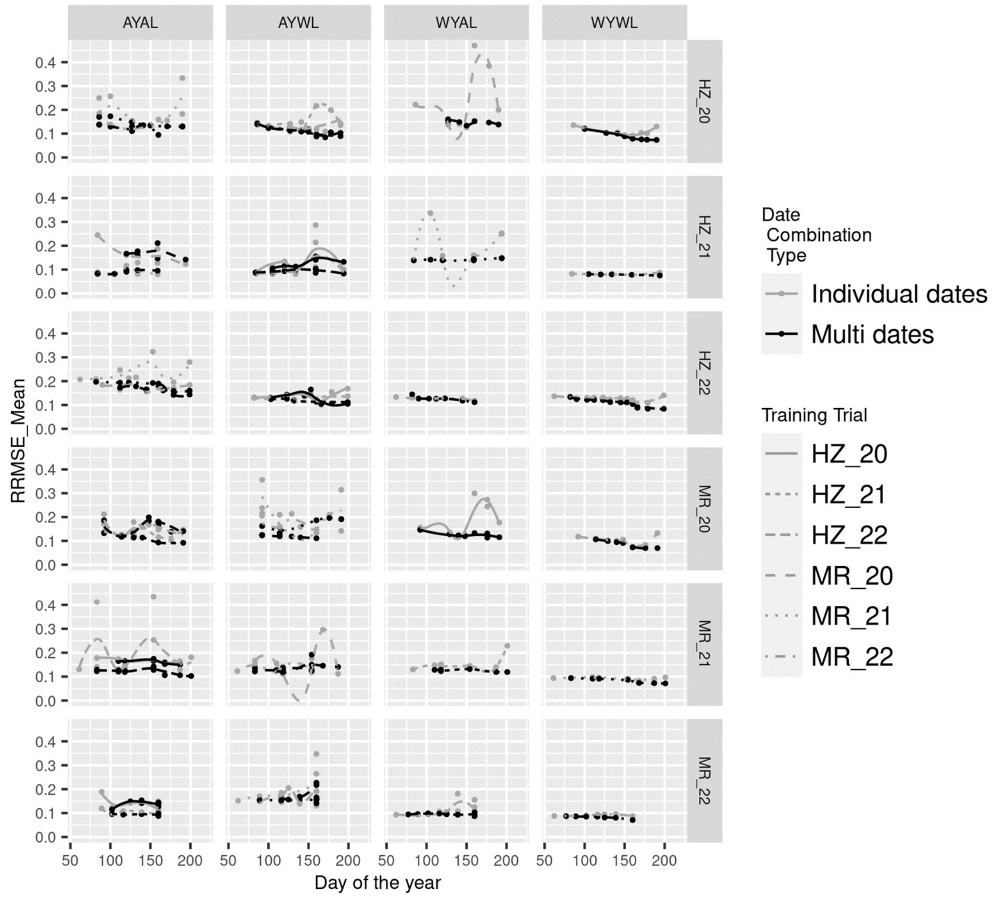

Figure S4. Comparison of model evaluation metrics by trial combination types (columns), date combination types (line colors), test trial (rows) and train trial (line types). W: within, A: across, Y: year, L: location. Lines are fitted by local polynomial regressions. For multi-date models (black), the metric values are positioned at the measurement date of the last included date. Thus, multi-date models include dates in the order of measurement. The full model is positioned at the rightmost position, respectively.

Table S1. Descriptive statistics of grain yield by field trial [g*m^{-2} for Mean, Min, Max and SD]. SD: standard deviation, CV: coefficient of variation. Groups were calculated by Tukey's post hoc test.

Trial	Mean	Min	Max	SD	CV	n	Groups
HZ_20	715	290	1031	108	15%	4349	d
HZ_21	818	500	1027	72	9%	2711	b
HZ_22	695	306	950	93	13%	1869	e
MR_20	792	223	1131	126	16%	4423	c
MR_21	849	300	1116	91	11%	2787	a
MR_22	714	350	927	70	10%	1785	d
